# Dual-Layer Composite Packaged FBG Sensor Array with Enhanced Thermal and Bending Performance for Extreme Engineering Environments

**DOI:** 10.3390/s26175525

**Published:** 2026-08-31

**Authors:** Zan Liu, Weijun Tong, Feng Wang, Qianfeng He, Shuhui Liu, Quanrong Deng, Wei Huang, Haoze Du

**Affiliations:** 1 Hubei Key Laboratory of Optical Information and Pattern Recognition, Wuhan Institute of Technology, Wuhan 430205, China; 22410010010@stu.wit.edu.cn (Z.L.); 22410010012@stu.wit.edu.cn (F.W.); 22510010013@stu.wit.edu.cn (Q.H.); liushuhui@wit.edu.cn (S.L.); quanrongdeng@wit.edu.cn (Q.D.); huangw@wit.edu.cn (W.H.); 2 Wuhan Fibersight Optoelectronic Science and Technology Corporation Ltd., Wuhan 430200, China

**Keywords:** Fiber Bragg grating, high-temperature stability, high-precision temperature measurement, bending performance

## Abstract

Aerospace and power equipment require precise temperature monitoring in extremely high-temperature environments, which often feature narrow and curved installation spaces. Since traditional thermocouples cannot provide both measurement performance and structural flexibility simultaneously, we propose a flexible fiber Bragg grating (FBG) temperature-sensing array. The sensor features a dual-layer composite packaging structure comprising an inner braided quartz-wool layer and an outer nickel-based alloy tube. Combined with a high-temperature annealing process, the packaged array was evaluated against a WRP-191 reference thermocouple. Over the investigated heating and cooling points up to 1000 °C, the maximum absolute indication error, max|T_FBG_ − T_ref_|, was 2.02 °C and the mean absolute indication error was 1.25 °C. These quantities are comparison errors relative to the reference thermocouple and are not expanded uncertainties. The dual-layer design also allowed a minimum demonstrated bending radius of 22 mm, supporting deployment in confined and curved installation paths.

## 1. Introduction

Aerospace and power generation are strategic emerging industries. The performance, efficiency, and safety of key equipment in these fields directly affect major engineering projects [1,2]. Currently, the internal working temperatures of these devices are constantly increasing to improve operating efficiency and output power. In such extreme environments, precise and real-time temperature monitoring is essential to optimize operating efficiency and predict structural fatigue in key components [3,4,5]. The internal working conditions of these devices are highly complex and often involve the coupling of multiple physical fields. Furthermore, the internal space is narrow, and the installation paths are curved [6,7,8,9]. These harsh conditions pose strict challenges for temperature sensors. Sensors must provide excellent high-temperature repeatability, high measurement accuracy, and fast response speeds. Additionally, they must have a flexible and bendable structure. Therefore, developing highly reliable temperature measurement technologies for extreme heat and curved spaces has great engineering and strategic significance.

In recent years, optical fiber sensing technology has attracted widespread attention. It offers unique advantages, including compact size, light weight, immunity to electromagnetic interference, and corrosion resistance. Additionally, it easily enables quasi-distributed measurements. In fiber-optic temperature-sensing technology, interferometric sensors (such as MZI and FPI) exhibit high measurement sensitivity but are difficult to multiplex to form sensor arrays. Sapphire fiber sensors offer exceptional temperature resistance, typically capable of withstanding temperatures up to 1600 °C over long periods; however, their manufacturing costs are high, and their packaging methods are complex. Distributed sensing technology possesses advantages in long-distance and wide-area spatial coverage, yet it suffers from relatively low spatial resolution and expensive interrogation systems [10]. In contrast, FBG sensing technology features comparatively lower costs and higher spatial resolution, enabling the formation of precise quasi-distributed sensing. Furthermore, its simple packaging process makes it highly suitable for deployment of complex equipment within fields such as aerospace and power generation. In 2011, Barrera et al. packaged a regenerated FBG using a ceramic tube and a nickel alloy shell [11]. This sensor can operate from room temperature to 1100 °C. It showed no significant wavelength drift. In the same year, Adamovsky et al. packaged a regenerated FBG using a double ceramic tube structure [12]. Its temperature measurement range was 20 °C to 800 °C. They tested it in the exhaust environment of a transport aircraft engine. The measurement trend matched that of electrical sensors, but the response was relatively slow. In 2015, Mamidi et al. packaged a femtosecond laser-inscribed FBG using an aluminum nitride capillary [13]. A stainless-steel tube provided external protection. This design achieved temperature measurement from 20 °C to 650 °C. The resolution was 1 °C, the linearity was 0.999, and the measurement error was ±1.23%. In 2024, Khan et al. encapsulated an FBG in a stainless-steel capillary [14]. They filled it with ceramic containing 0% to 50% iron powder. They found that 40% iron powder increased the temperature sensitivity to 14.63 pm/°C. It also shortened the response time to 180 s and provided an estimated lifespan of 4.46 years. However, few of these traditional packaging strategies address the balance between robust high-temperature protection and structural flexibility, leaving a gap in sensors capable of navigating curved and narrow pathways in practical engineering applications.

In this paper, we implement and experimentally characterize a flexible FBG array with a dual-layer composite package comprising a braided quartz-wool inner layer and a GH3030 outer tube. The contribution is an implementation-specific extension of the general compliant-inner/protective-outer packaging concept to a four-grating high-temperature sensing array, rather than a claim that the general packaging concept is itself new. A high-temperature-resistant FBG array with four wavelengths (1524, 1534, 1544, and 1554 nm) was fabricated using femtosecond laser direct writing. A 950 °C cyclic annealing process was applied, reducing the wavelength drift to below 5 pm/h, thereby improving the measurement accuracy in high-temperature environments. The inner braided quartz wool protects the optical fiber and restricts its movement, enabling adaptation to narrow and curved spaces, whereas the outer nickel-based alloy tube provides mechanical protection and bending capability. Calibration tests established a temperature-wavelength fitting function with a correlation coefficient greater than 0.9999. The maximum absolute indication error was 2.02 °C, the mean absolute error over the tested heating and cooling points was 1.25 °C, and the minimum demonstrated bending radius was 22 mm. The measured T99 response time was 144.17 ± 2.75 s. During 48 h output monitoring at an 800 °C furnace setpoint, the three sensor outputs exhibited peak-to-peak variations of 3.89, 4.33, and 3.58 °C, respectively, with a mean peak-to-peak value of 3.93 °C. Because no independent reference temperature was recorded continuously over the same interval, this result characterizes total output variation under the furnace setpoint rather than intrinsic sensor drift. Rather than establishing universal superiority, these results demonstrate an implementation-specific combination of high-temperature operation, measurement performance, and deployment flexibility, with the reference cases and metric limitations stated explicitly in Section 4.2 and Table 3.

## 2. Sensing Mechanism of the High-Temperature FBG Array

The sensing principle of the FBG is based on the Bragg diffraction condition. A broadband light beam is injected into the optical fiber. Light satisfying the Bragg condition is then reflected. This creates a narrow-band reflection peak in the reflection spectrum. According to the coupled-mode theory, the central wavelength of the reflected light is expressed as follows:

(1) $$\lambda_{B}=2{\cdot n}_{eff}\cdot \Lambda$$

Here, $n_{eff}$ represents the effective refractive index of the fiber core. Λ represents the grating period. External physical fields can act on the FBG. When this happens, both Λ and $n_{eff}$ change accordingly. Consequently, the central wavelength $\lambda_{B}$ shifts. The change in physical quantities can be detected by demodulating this reflected central wavelength. When only the ambient temperature changes, the thermal expansion effect alters the grating period. This change is expressed as:

(2) ΔΛ=α⋅Λ⋅ΔT

Here, *α* represents the thermal expansion coefficient of the fiber grating. Δ*T* represents the change in ambient temperature. The thermo-optic effect alters the effective refractive index. This change is expressed as:

(3) $$\Delta n_{eff}={\beta \cdot n}_{eff}\cdot \Delta T$$

Here, β represents the thermo-optic coefficient of the fiber grating, and its total response can be expressed as:

(4) $$\Delta \lambda_{B}=\lambda_{B}\cdot [(\alpha +\beta )\cdot \Delta T]$$

At room temperature, the thermal expansion coefficient α and the thermo-optic coefficient β are constant. However, at high temperatures, both coefficients become nonlinear functions of temperature [15]. The function of the grating period with respect to temperature can be expressed as:

(5) $$\Lambda (T)=\Lambda_{0}\cdot (1+a\cdot T+b\cdot T^{2})$$

where a represents the first-order coefficient of thermal expansion, and b represents the second-order coefficient of thermal expansion. The effective refractive index as a function of temperature can be expressed as:

(6) $$n_{eff}(T)=n_{0}+c\cdot T+d\cdot T^{2}$$

The FBG central wavelength as a function of temperature, retaining up to the third-order term, can be expressed as:

(7) $$\lambda_{B}(T)\approx \lambda_{0}[1+(a+\frac{c}{n_{0}})\cdot T+(b+\frac{d+a\cdot c}{n_{0}})\cdot T^{2}+(\frac{a\cdot d+b\cdot c}{n_{0}})\cdot T^{3}]$$

Combining and simplifying yields:

(8) $$\lambda_{B}(T)\approx \lambda_{0}+A\cdot T+B\cdot T^{2}+C\cdot T^{3}$$

where $\lambda_{0}$ represents the central wavelength of the FBG at 0 °C, A represents the first-order coefficient, B represents the second-order coefficient, and C represents the third-order coefficient.

## 3. Packaging and Annealing of the High-Temperature FBG Array

**Figure 1:**
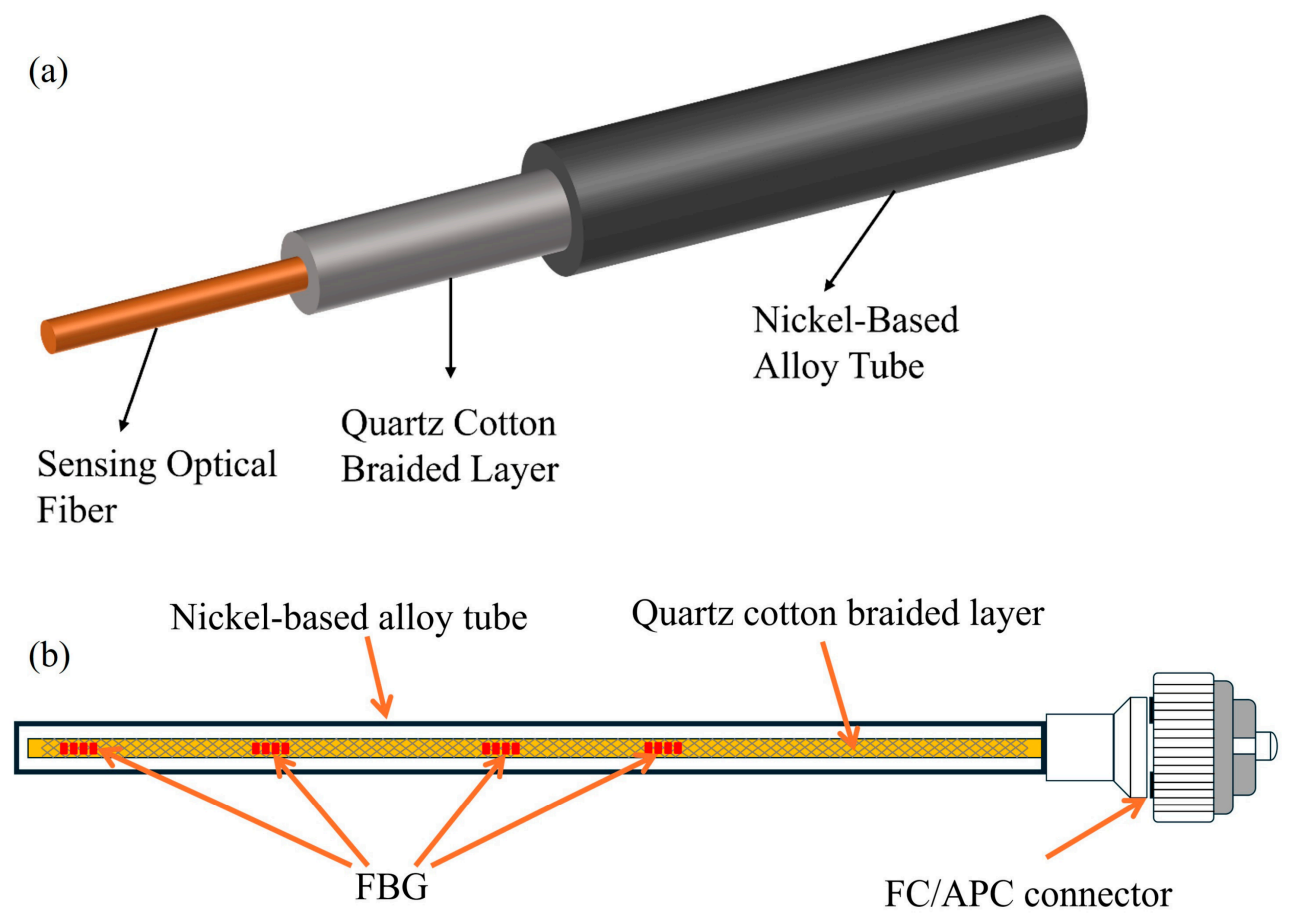
(**a**) 3D schematic of the packaging structure. (**b**) 2D schematic of the packaging structure.

**Figure 2:**
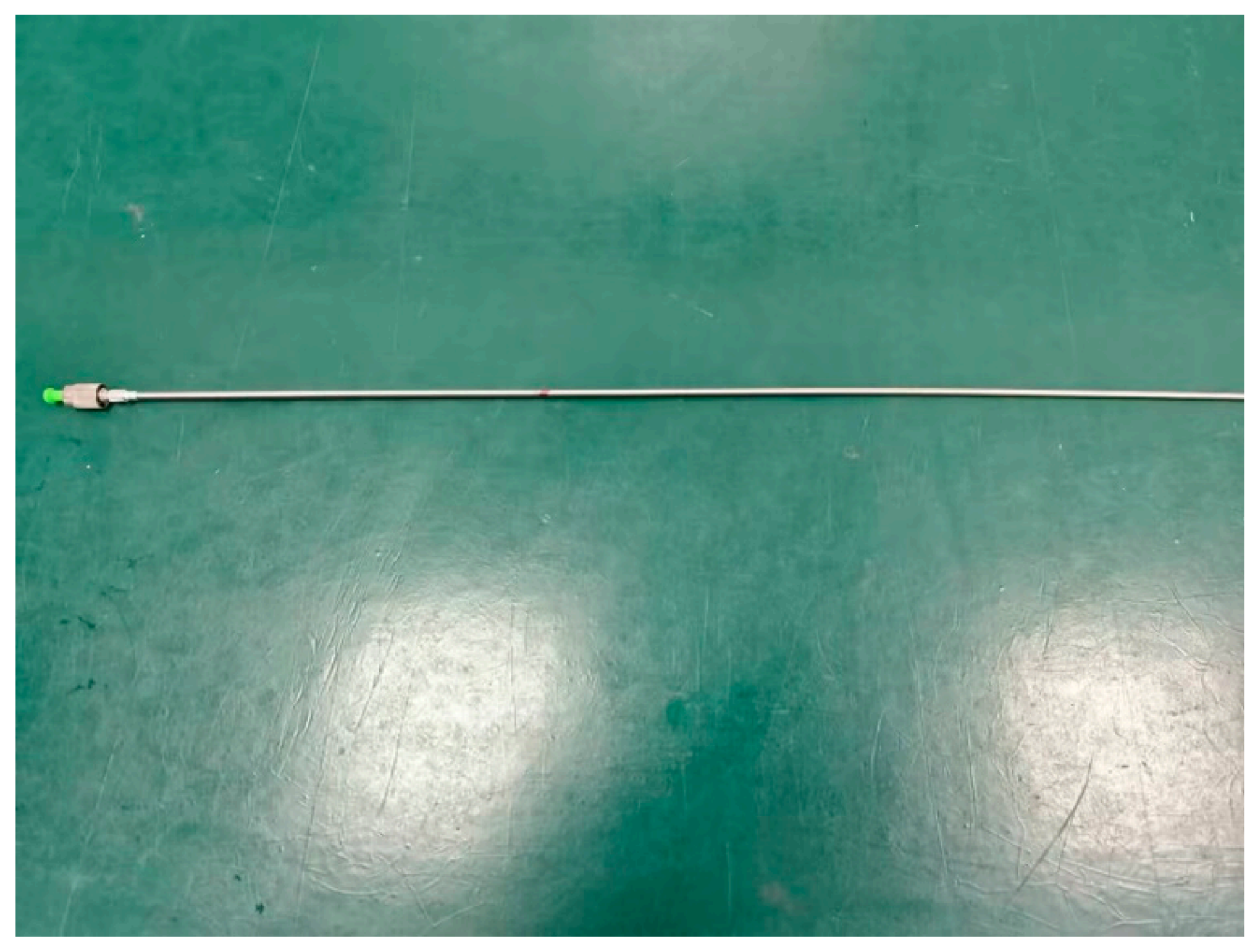
Photograph of the fabricated sensor.

In practical engineering applications, optical fibers and their grating regions are highly susceptible to extremely harsh environments; therefore, reliable protective measures must be implemented [16]. In this study, polyimide-coated optical fibers were used for FBG inscription, and the sensor array was encapsulated in a tubular structure using high-temperature-resistant materials. A GH3030 nickel-based alloy tube was selected as the encapsulation shell. This material retains excellent mechanical strength at high temperatures up to 1000 °C and exhibits good plasticity after annealing, allowing it to be bent into U- or V-shapes at room temperature to accommodate complex installation scenarios. The interior of the alloy tube was filled with a braided quartz wool layer, which restricts the movement of the optical fiber to minimize bend-induced stress and enhance its overall bending performance. A schematic of the encapsulation structure is shown in Figure 1. Four FBGs were encapsulated within the tube in a free state, effectively preventing external mechanical stress from interfering with the measurements. The pigtail end of the optical fiber was terminated with an FC/APC connector to link with an external interrogator. For practical deployment, the number of FBGs in the array can be flexibly adjusted, and their spatial spacing can be customized based on the spatial resolution requirements of the target being measured. A photograph of the fabricated sensor is shown in Figure 2.

The polyimide coating is retained during femtosecond inscription and packaging. Fabrication nevertheless requires the manual placement of long quartz-wool and GH3030 sections, followed by assembly and high-temperature annealing. GH3030 is expected to be a principal material-cost contributor. No quantitative comparison of material cost, labor time, fabrication yield, or scale-up performance was conducted; therefore, cost-effectiveness and manufacturability relative to alternative packages are not claimed here.

The high-temperature stability of an FBG is a crucial factor for achieving high-precision temperature monitoring. In long-term high-temperature environments, the central wavelength of an FBG experiences a continuous shift, which compromises its repeatability and accuracy. Residual stress is introduced into the optical fiber during the rapid cooling phase of the manufacturing process, and the multi-photon absorption effects during femtosecond laser inscription also introduce residual stress at the microscale. Under high temperatures, the presence of these residual stresses alters the effective refractive index of the fiber, resulting in a significant drift in the central wavelength [17]. Annealing the FBG can effectively mitigate this wavelength drift, thereby ensuring high-precision temperature monitoring.

**Figure 3:**
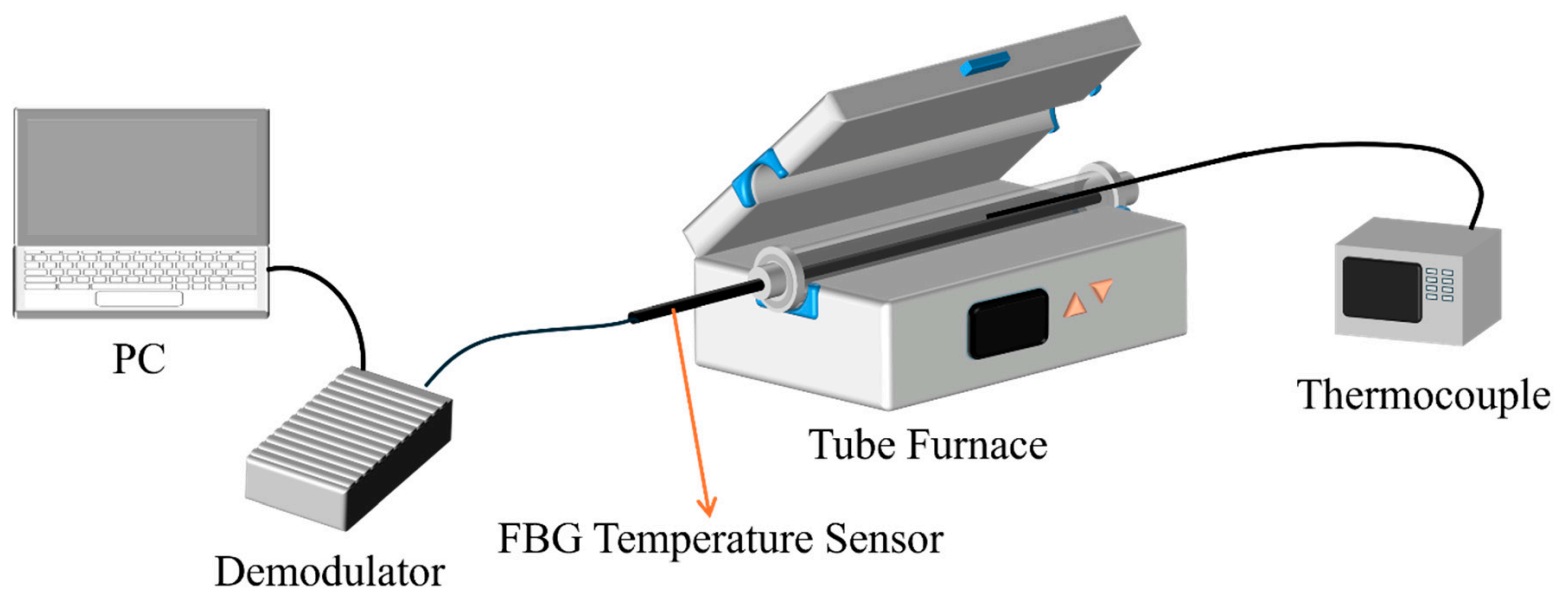
Schematic of the experimental setup.

A high-temperature thermal annealing experiment was conducted on the femtosecond laser-inscribed FBG sensor array; the experimental setup is illustrated in Figure 3. The tested FBG sensor array was placed inside the constant-temperature zone of a tube furnace (with a maximum operating temperature of 1200 °C) (SZGL-1200C, SIOMM, Shanghai, China) for heating. An S-type thermocouple (WRP-191, Yancheng Yijiu Electrical Appliances& Instruments, Yancheng, Jiangsu, China) served as the temperature reference to acquire the actual annealing temperature inside the furnace in real time. The FBG sensor array was connected to an FBG interrogator (Scan-S1040, Wuhan Wisfiber Technology, Wuhan, Hubei, China) to continuously monitor the dynamic changes in the reflection spectrum throughout the entire annealing process.

**Figure 4:**
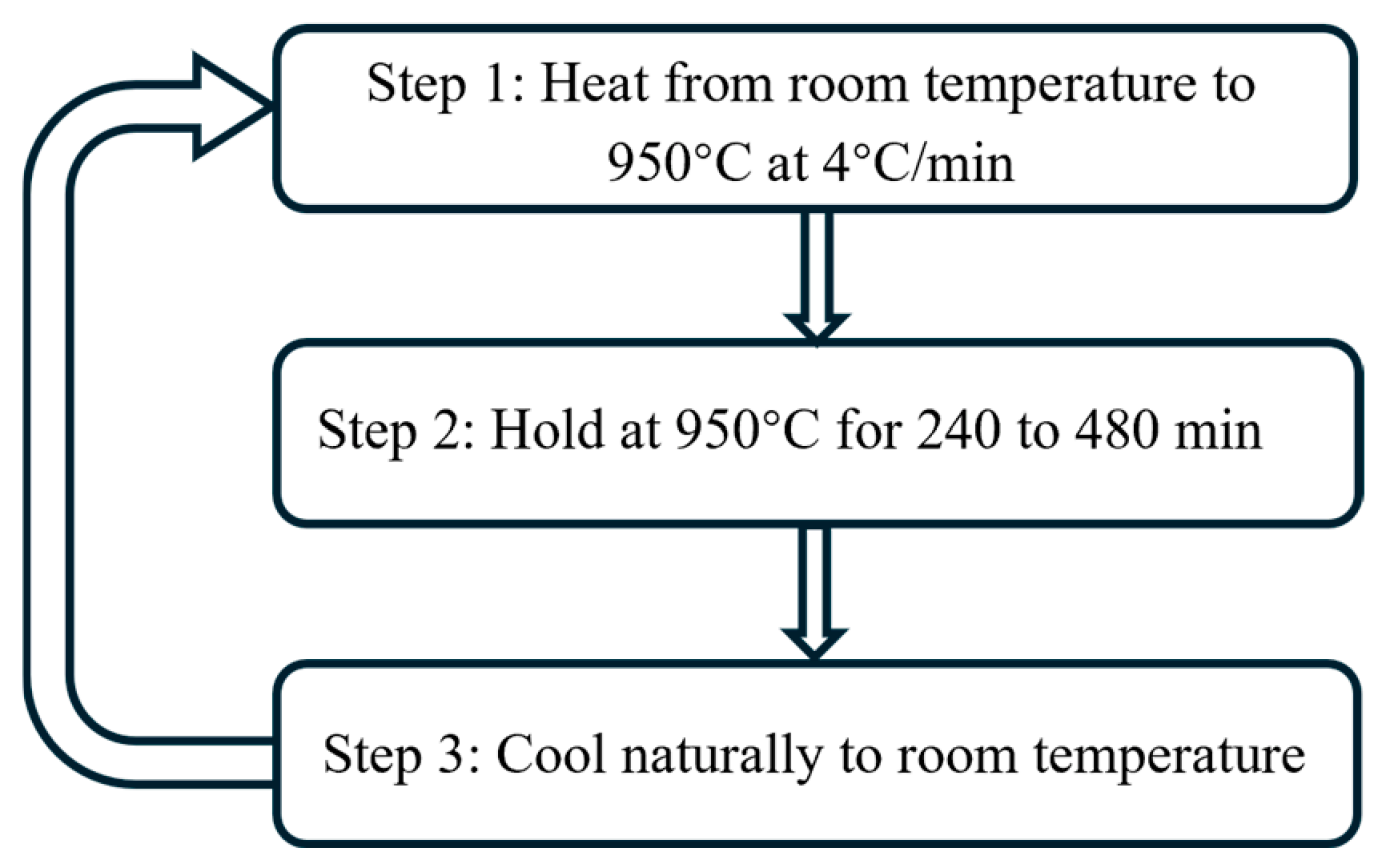
The annealing process.

The high-temperature annealing process in this experiment was divided into three stages, as shown in Figure 4. In the first stage, the temperature was slowly raised from room temperature to 950 °C at a heating rate of 4 °C/min. In the second stage, the temperature was maintained at 950 °C for 240 to 480 min. The specific duration was determined by the wavelength drift rate: when the drift rate was relatively low, the duration was set to 240 min. In the third stage, the system was naturally cooled from 950 °C down to room temperature. These three stages constituted one complete annealing cycle, which was repeated at least three times. Annealing was considered successful if the drift rate stayed below 5 pm/h for the last 3–4 h.

Setting the annealing temperature to 950 °C strikes a balance between improving annealing efficiency (thereby reducing the total annealing time) and minimizing the degradation of spectral performance. For FBGs inscribed in SMF-28 fiber, the annealing temperature must not exceed 1000 °C. At the same time, annealing at a lower temperature, such as 800 °C, for a longer duration will also result in some loss of spectral performance. In summary, an annealing temperature of 950 °C achieves an optimal compromise between shortening the annealing time and mitigating spectral performance loss.

**Figure 5:**
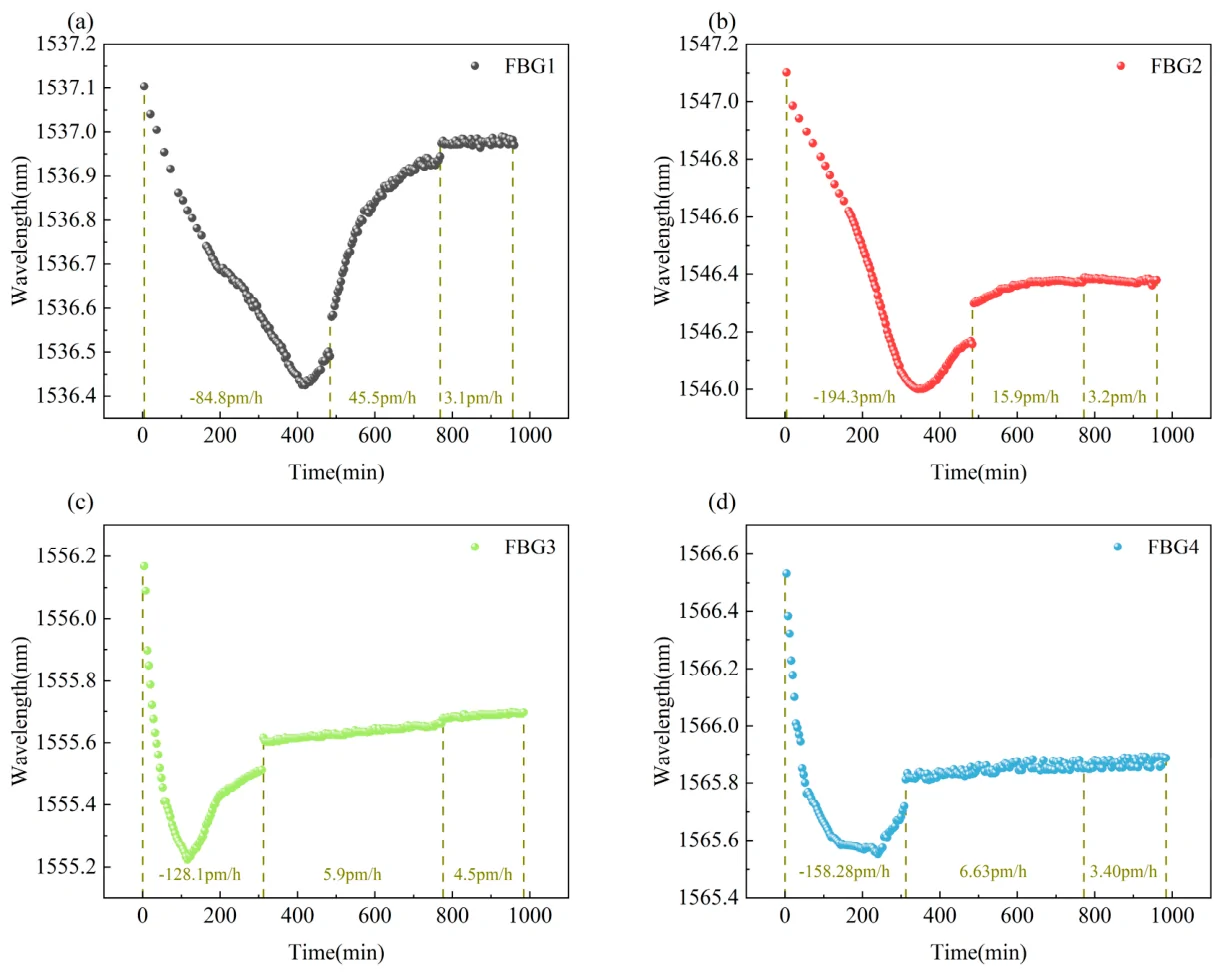
Wavelength variations of FBGs with different initial wavelengths under high-temperature annealing at 950 °C: (**a**) FBG1 with a wavelength around 1537 nm at 950 °C; (**b**) FBG2 with a wavelength around 1547 nm at 950 °C; (**c**) FBG3 with a wavelength around 1556 nm at 950 °C; (**d**) FBG4 with a wavelength around 1566 nm at 950 °C.

Figure 5 illustrates the wavelength shifts of fiber Bragg gratings (FBGs) with varying central wavelengths during the annealing process at 950 °C. The FBGs exhibit consistent drift trends at this temperature, regardless of their initial central wavelengths. During the first annealing cycle, the central wavelength decreases rapidly at a rate of up to 194.3 pm/h, subsequently rising after reaching its minimum value. This phenomenon can be attributed to the carbonization, disintegration, and ultimate detachment of the polyimide coating in environments exceeding 300 °C. Specifically, during the initial slow temperature ramp-up, the polyimide coating carbonizes. As the temperature continues to rise, the optical fiber experiences gradual axial thermal expansion. The mismatch in the coefficients of thermal expansion (CTE) between the carbonized polyimide and the silica fiber causes the coating to gradually delaminate. During the isothermal holding phase at 950 °C, the residual stress inherent from the fiber manufacturing process is relieved, and the residual coating continues to shed until complete detachment occurs. At this critical point, the wavelength shift reaches its minimum on the curve. In subsequent cycles, the initial wavelength of each cycle is significantly higher than the final wavelength of the preceding cycle. This indicates that the natural cooling phase also facilitates residual stress relaxation at an accelerated rate. Over the course of the three cycles, prolonged exposure to 950 °C continuously and gradually relieves the internal residual stress of the optical fiber. Ultimately, the wavelength drift progressively decelerates until it achieves a stable state (<5 pm/h) at 950 °C.

**Figure 6:**
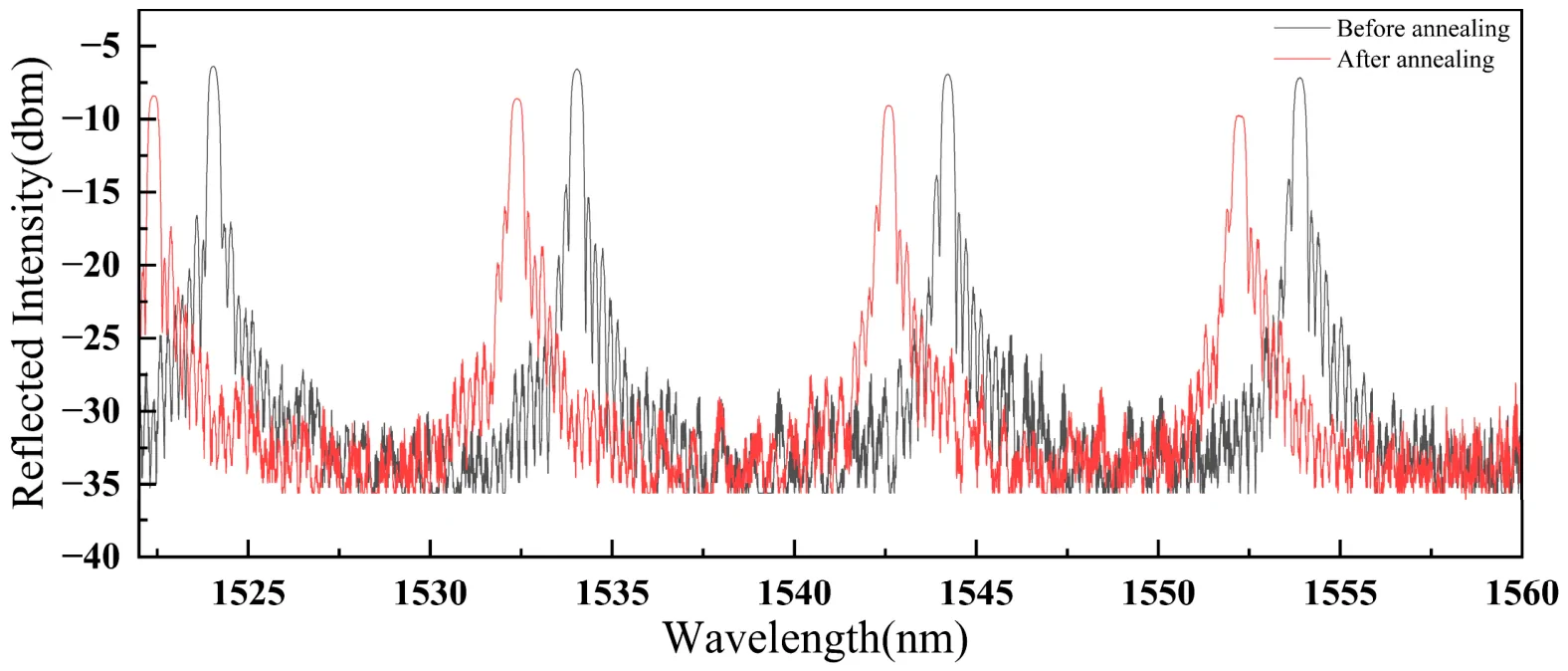
Optical spectra of the FBG array at room temperature before and after annealing.

Figure 6 illustrates the room-temperature optical spectra of the FBG array before and after annealing. Prior to annealing, the central wavelengths of FBG1 to FBG4 were 1524.04, 1534.04, 1544.21, and 1553.89 nm, respectively. Post-annealing, these values shifted to 1522.39, 1532.40, 1542.61, and 1552.21 nm. Consequently, all four peaks exhibited a significant blue shift, moving toward the shorter wavelength region by an average of approximately 1.65 nm. Concurrently, the intensity of the reflection peaks experienced a slight reduction, indicating that the high-temperature treatment induced a minor degradation in grating reflectivity. Although extending the annealing time can further reduce the wavelength drift rate, it also degrades the spectral performance. Nevertheless, the optical spectrum of the annealed FBG array maintains a high signal-to-noise ratio (SNR), ensuring accurate peak wavelength demodulation. This demonstrates the array’s robust sensing performance in high-temperature environments.

The non-uniform wavelength drift observed after annealing is primarily attributed to differences in the initial stress of the FBGs during the packaging process, variations in the release of residual stress during annealing, and the temperature gradient within the tube furnace. In our experiments, the initial spectral spacing between the gratings was approximately 10 nm. The maximum difference in wavelength drift caused by annealing was 1.64 nm. At 1000 °C, the maximum wavelength drift of individual gratings reached 14.46 nm, with a maximum differential wavelength drift of 0.19 nm. The maximum spectral bandwidth of the gratings at high temperatures was 1.95 nm.

When the temperature of the measurement region is between 900 °C and 1000 °C, the minimum remaining wavelength division multiplexing (WDM) margin is 4.43 nm, which is sufficient to prevent spectral overlaps or crosstalk. However, when the temperature of the measurement region spans from 600 °C to 1000 °C, the minimum remaining WDM margin drops to −0.47 nm, which could lead to spectral overlaps or crosstalk under extreme conditions. Therefore, when the temperature variation in the measurement region spans across the 600–1000 °C range, the initial spectral spacing should be increased to 11 nm or 12 nm. For a broader measurement range from 0 °C to 1000 °C, the initial spectral spacing should be increased to 19 nm.

For larger-scale arrays containing a greater number of sensing elements, the following strategies can be employed: (1) preemptively allocating spectral spacing based on the expected temperature range of the measurement zones; (2) further optimizing the annealing process to minimize the disparity in wavelength drift; and (3) combining WDM with Spatial Division Multiplexing (SDM).

## 4. Performance Characterization of the High-Temperature FBG Array

To verify the measurement accuracy and structural flexibility resulting from the proposed packaging structure and annealing process, a series of tests were conducted to characterize the performance of the FBG sensor [18], including calibration, accuracy, response time, repeatability, and bending tests.

### 4.1. Calibration of the FBG Sensor

**Figure 7:**
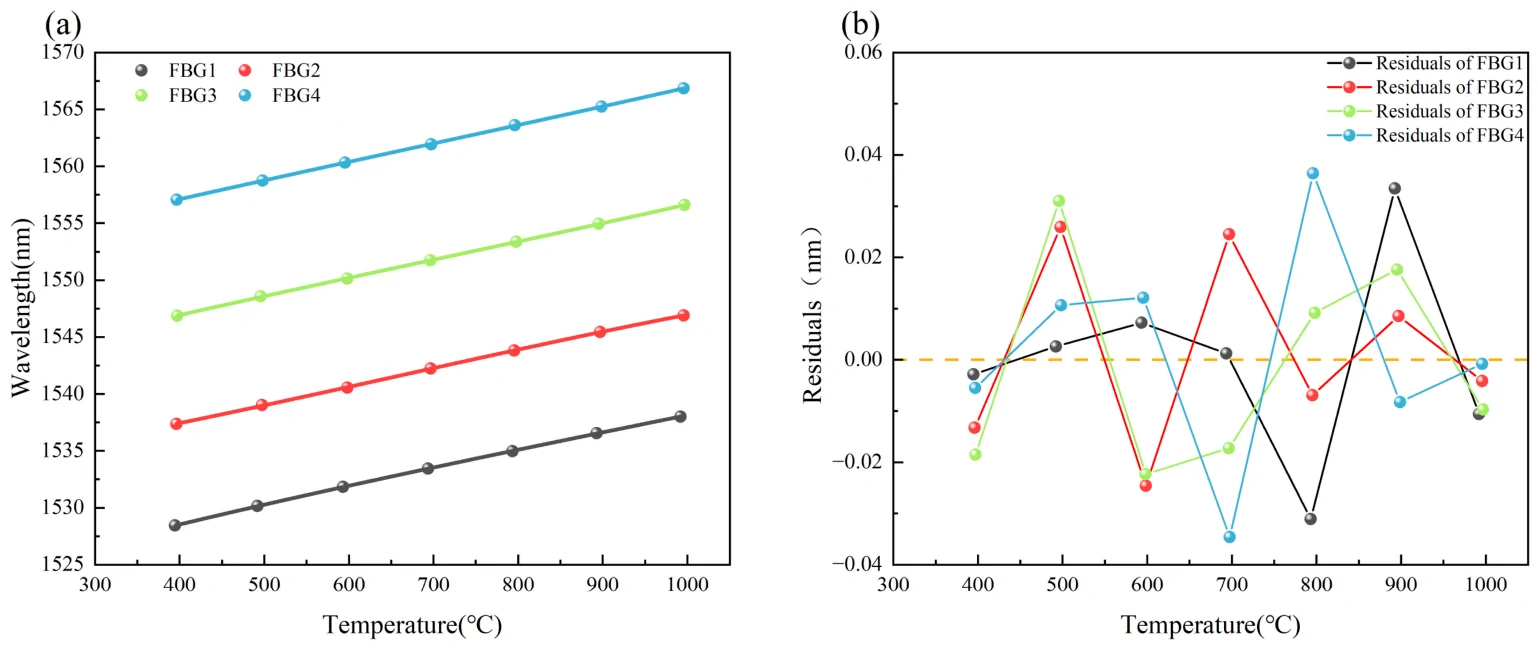
(**a**) Central-wavelength-temperature calibration curves. (**b**) In-sample residuals at the seven calibration points. Error bars denote the standard deviation of the 1 s central-wavelength samples acquired during each 30 min isothermal hold.

Temperature calibration was performed from 400 to 1000 °C using seven calibration points at 100 °C intervals. Thermocouple probes were collocated with the grating positions. At each point, the central wavelength and reference temperature were recorded at 1 s intervals during a 30 min isothermal hold and time-averaged. The error bars in Figure 7b and the values in Table 1 are the standard deviations of the central-wavelength samples acquired during those holds. A cubic polynomial was fitted to each FBG in accordance with the nonlinear high-temperature response described by Equation (8). Because four coefficients are estimated from seven calibration points, only three residual degrees of freedom remain; the calibration residuals are therefore treated as an in-sample diagnostic and not as proof against overfitting.

(9) $$\lambda_{FBG1}(T)=1520.5568+0.02252T-7.4139\times 10^{-6}T^{2}+2.4826\times 10^{-9}T^{3}$$

(10) $$\lambda_{FBG2}(T)=1532.5336+0.008371T-1.2235\times 10^{-5}T^{2}+6.1689\times 10^{-9}T^{3}$$

(11) $$\lambda_{FBG3}(T)=1539.6040+0.02041T-6.4004\times 10^{-6}T^{2}+3.0449\times 10^{-9}T^{3}$$

(12) $$\lambda_{FBG4}(T)=1549.9862+0.01931T-4.6178\times 10^{-6}T^{2}+2.2552\times 10^{-9}T^{3}$$

The cubic fits have correlation coefficients greater than 0.9999, and Figure 7b shows the in-sample residuals at the seven calibration points. The residual distribution is consistent with the fitted curves over the calibration range, but the small number of residual degrees of freedom does not establish model uniqueness or exclude overfitting. Accordingly, model performance is assessed separately at the five intermediate validation temperatures described in Section 4.2, and no claim of universal cubic-model adequacy is made.

### 4.2. Indication-Error Evaluation Against the Reference Thermocouple

After calibration, measurements at 500 to 1000 °C were compared with simultaneous WRP-191 reference indications at 50 °C intervals during two heating and cooling cycles. The five intermediate points (550, 650, 750, 850, and 950 °C), which were not used in the 100 °C-spaced calibration fit, constitute the independent validation temperatures. Calibration-point residuals in Figure 7b are therefore distinct from prediction errors at these intermediate points. The retained analysis record supports the aggregate paired-observation statistics reported below, but it does not preserve the complete pointwise per-FBG validation predictions required to reconstruct separate RMSE, bias, and maximum absolute error values for every FBG. Those unverified per-FBG statistics are not inferred from the plotted figure, and this limitation is disclosed explicitly.

**Figure 8:**
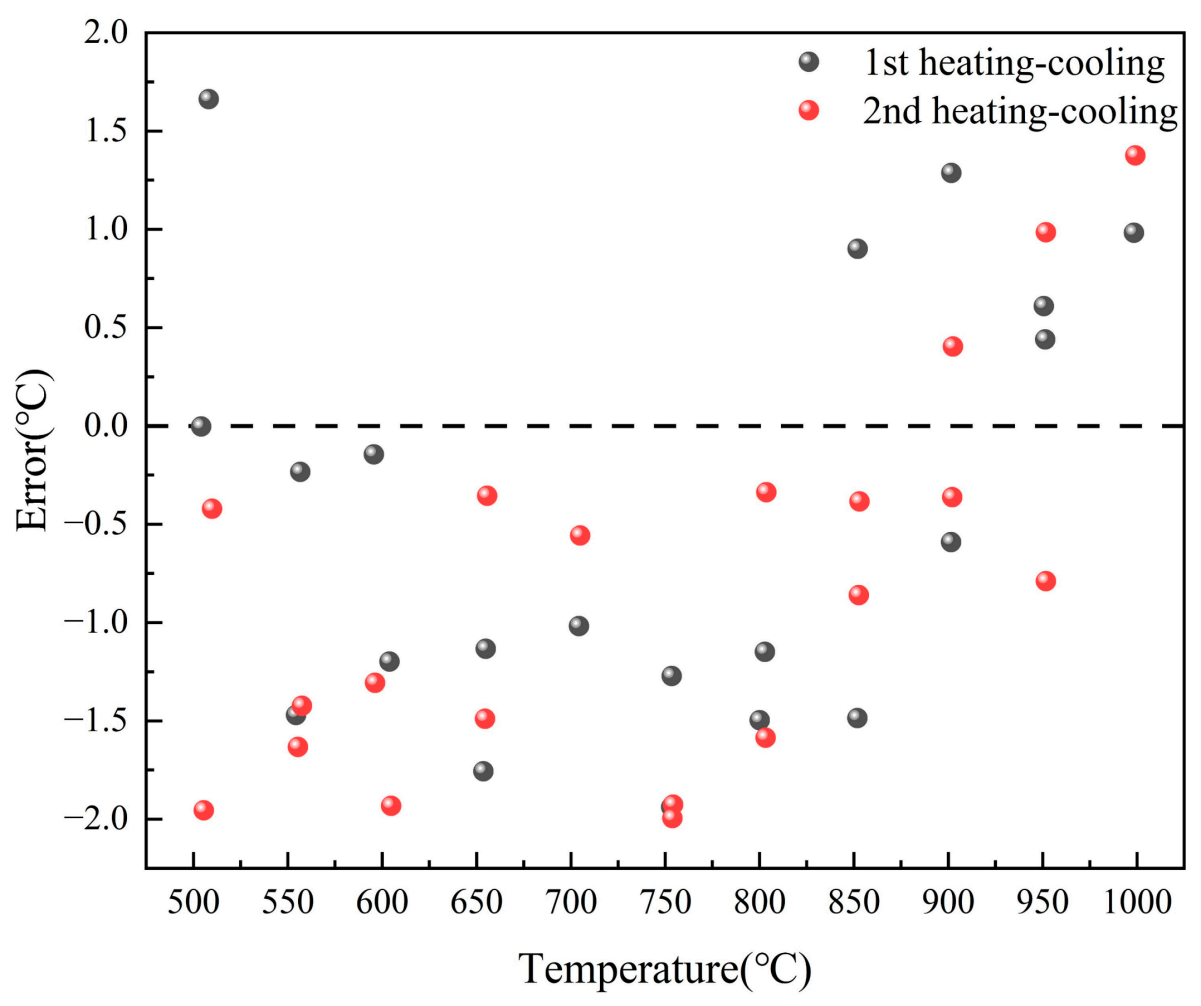
Indication errors of the FBG array relative to the WRP-191 reference thermocouple.

For each paired observation, the indication error was defined as e = T_FBG_ − T_ref_, where T_FBG_ is the temperature obtained from the FBG calibration function, and T_ref_ is the simultaneously recorded WRP-191 reference indication. As shown in Figure 8, the maximum absolute indication error, max|e|, over the two heating and cooling cycles was 2.02 °C, and the mean absolute indication error, mean (|e|), was 1.25 °C. These statistics quantify agreement with the reference indications; they are not absolute measurement accuracy, combined standard uncertainty, or expanded uncertainty. The WRP-191 documentation available for this study states a tolerance of 0.2% FS + 0.5 °C. However, the configured full-scale value, calibration-certificate uncertainty, probability distribution, and degrees of freedom required for a defensible uncertainty propagation were not documented in the experimental record. Accordingly, the tolerance statement has not been converted into a standard uncertainty or combined by root-sum-square with the observed indication errors. No confidence level, coverage factor, or expanded uncertainty is assigned to the values 2.02 and 1.25 °C. Table 2 summarizes the definitions and statistical status of the reported quantities.

Table 3 provides a descriptive comparison of the temperature range, error metric, bending radius, and response time reported for each sensor. The values have not been normalized to 1000 °C. Instead, each error entry is stated with its source-specific temperature range or evaluation condition. NR denotes that a directly comparable value was not reported or that the applicable temperature basis could not be verified from the source information retained for this comparison. For the present work, the mean absolute indication error of 1.25 °C and maximum absolute indication error of 2.02 °C were calculated over two heating and cooling cycles from 500 to 1000 °C and are not values specifically measured at 1000 °C. The Kumar result is limited to its reported range up to 900 °C, and the Khan range ends at 700 °C. Because the studies differ in temperature range, reference standard, experimental protocol, and metric definition, Table 3 is not used to establish a direct accuracy ranking or universal performance superiority.

For bending capability, the source-reported case in Table 3 is the flexible INFIBRA sensor with a minimum bending radius of 150 mm, whereas the present four-grating package remained spectrally demodulatable at a bending radius of 22 mm. For response time, the T99 value of 144.17 ± 2.75 s (1 s sampling; n = 3) for the present sensor lies between the values reported by Barrera et al. (50 s), Khan et al. (180 s), and Stadler et al. (250 s). These comparisons describe the reported values only; differences in endpoint definitions and test conditions prevent a strict ranking. The table therefore indicates application-specific trade-offs among bending capability, indication error, operating range, and response time.

### 4.3. Response Time Testing of the FBG Sensor

The FBG sensor and WRP-191 thermocouple were collocated in the tube furnace. Response time was evaluated using a common T99 endpoint: elapsed time from the start of a temperature transition until the output first reached 99% of the total change relative to the final plateau. For each FBG trace, the final plateau was the mean output over the last 30 s. The FBG interrogator sampled at 1 s intervals. During nine stepwise comparisons from 510 to 800 °C in 30 °C increments, the thermocouple was recorded at 60 s intervals; consequently, thermocouple-based response differences have a coarse temporal resolution of approximately one 60 s sample.

Using the T99 criterion, the FBG reached the endpoint 120 ± 40 s earlier than the thermocouple across nine stepwise comparisons. Because of the 60 s thermocouple sampling interval, this value is a coarse comparative estimate rather than a high-resolution response-time difference. In three room-temperature-to-950 °C transfer tests evaluated from the 1 s FBG records using the same T99 rule, the FBG response time was 144.17 ± 2.75 s (mean ± standard deviation).

**Figure 9:**
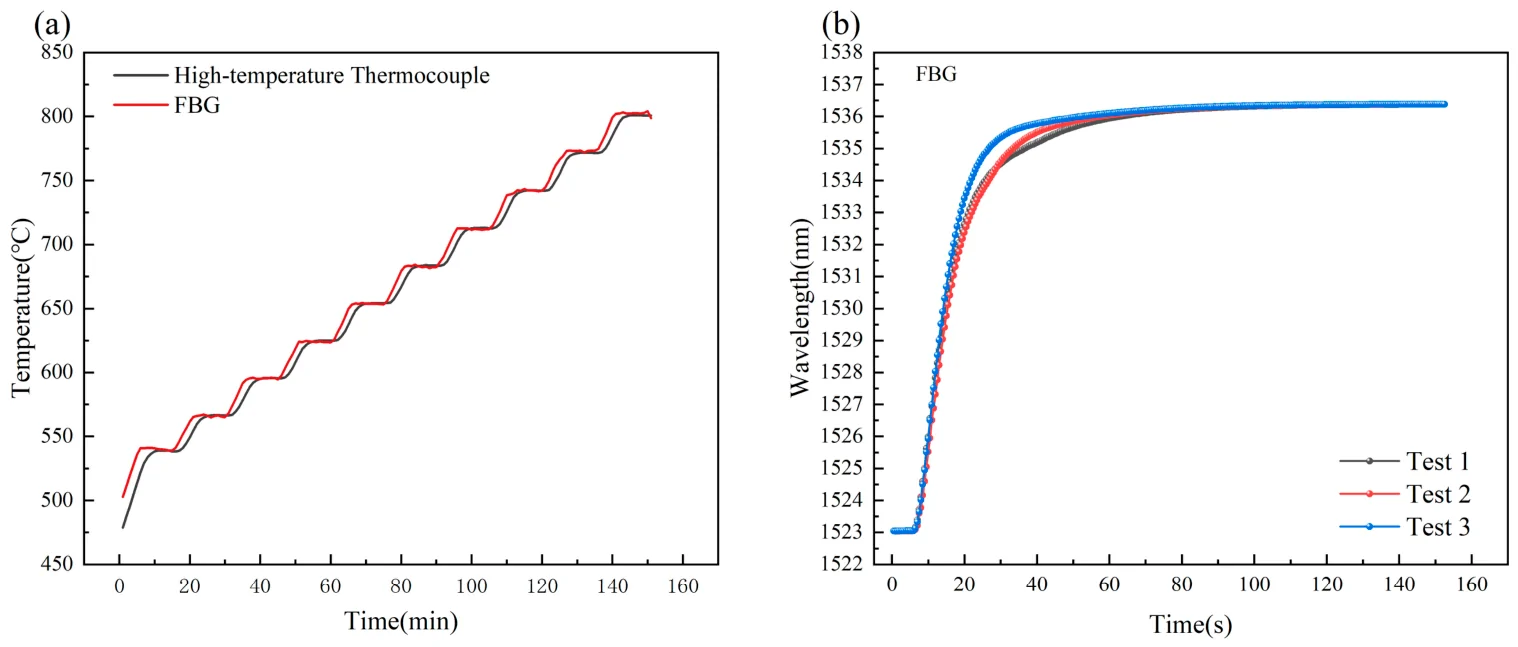
(**a**) Nine stepwise response comparisons using the T99 endpoint; thermocouple sampling interval: 60 s. (**b**) Three FBG room-temperature-to-950 °C transfer tests using T99 and 1 s sampling.

Figure 9 and Table 4 summarize the response comparison and sensor construction. Under the stated T99 rule, the FBG reached the endpoint earlier than the WRP-191 in the nine stepwise tests, but the 60 s thermocouple sampling interval limits the precision of the reported 120 ± 40 s difference. The three FBG transfer tests provide the higher-resolution 144.17 ± 2.75 s result from 1 s records. The faster observed response is consistent with differences in package thermal transport and thermal mass: alumina conductivity decreases to approximately 7 W/(m·K) at 800 °C [23], whereas GH3030 reaches 25.1 W/(m·K), the specific values are listed in Table 5, and the quartz wool and optical fiber have small thermal masses. These mechanisms are interpretive; the response values remain specific to the stated geometry, endpoint, and test protocol.

### 4.4. Repeatability Testing of the FBG Sensor

**Figure 10:**
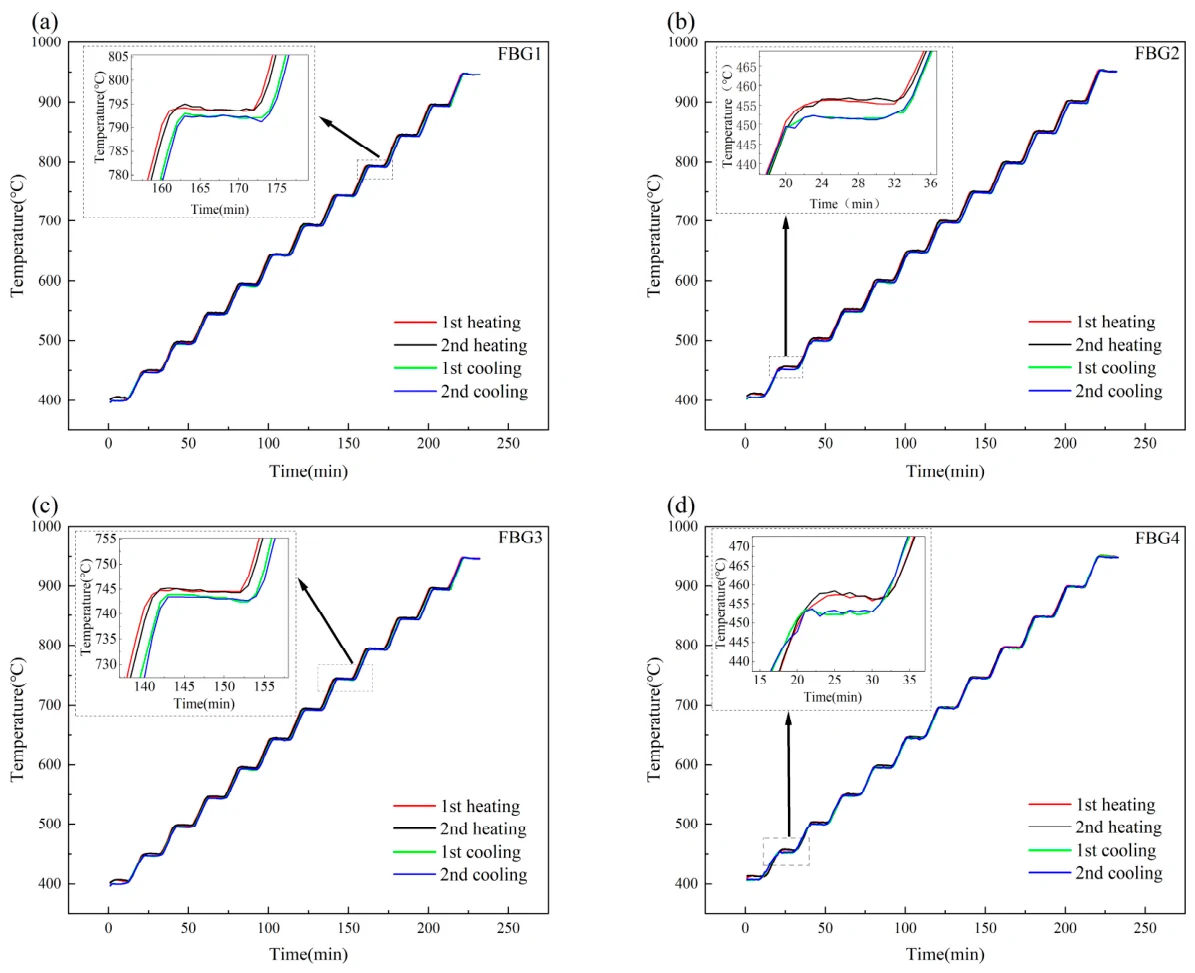
Repeatability test of the FBG sensor. Comparison of heating and cooling repeatability for (**a**) FBG1; (**b**) FBG2; (**c**) FBG3; (**d**) FBG4.

**Figure 11:**
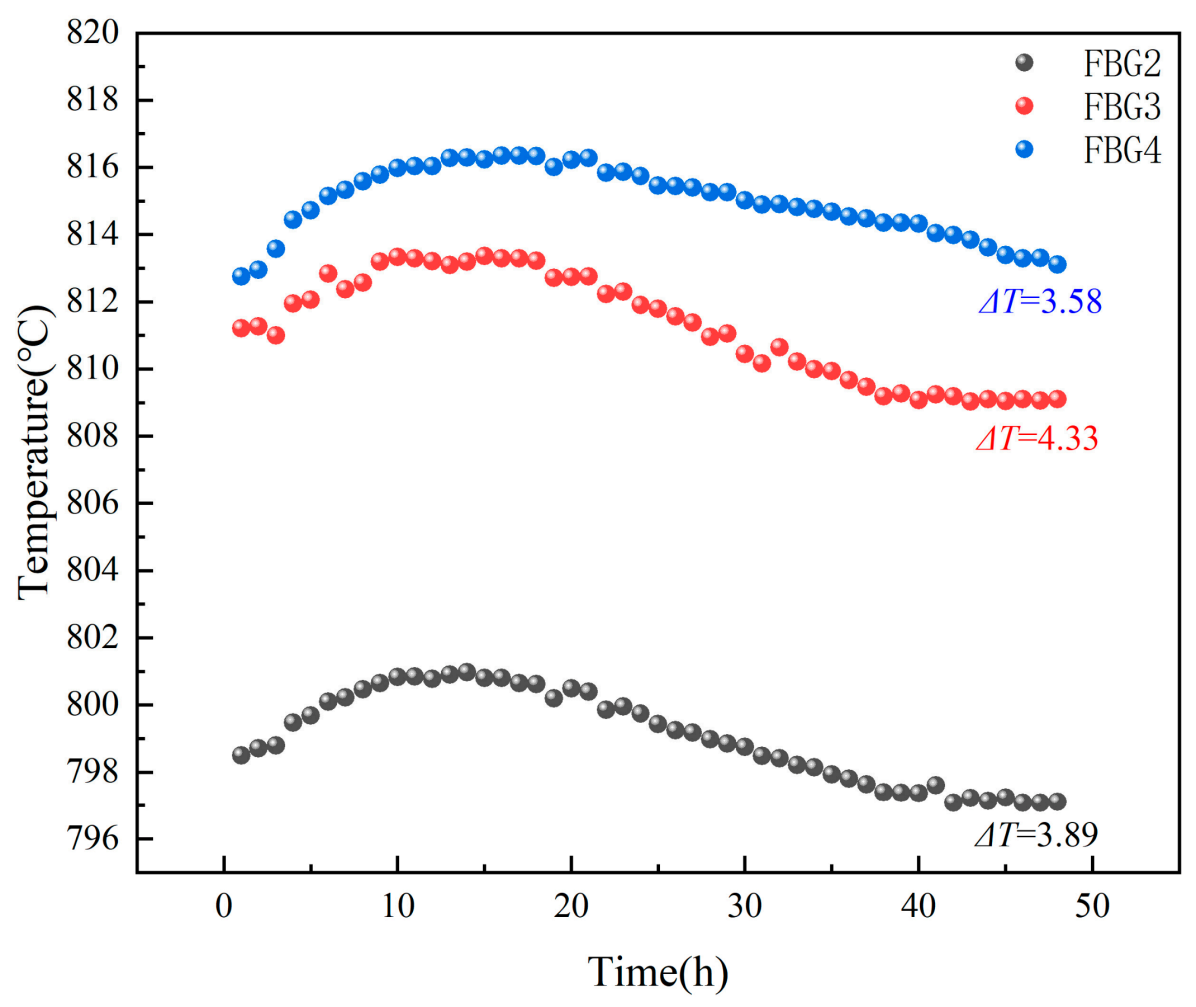
Temperature outputs of the three sensors during 48 h monitoring at an 800 °C furnace setpoint.

Sensor repeatability is a critical metric for evaluating practical engineering applicability. Thermal cycling tests ranging from 400 °C to 1000 °C were conducted on each sensor within the array. The thermal profile was programmed in 50 °C increments, with each step comprising an 8-min transient ramp-up phase followed by a 12-min isothermal holding phase. The cooling process adhered to an identical temperature variation rate and holding duration. Throughout both the heating and cooling phases of each cycle, the response data were continuously recorded by an optical interrogator for comparative analysis, as illustrated in Figure 10. In addition, three sensors were randomly selected for 48 h output monitoring at a furnace setpoint of 800 °C, as shown in Figure 11. An independent reference-thermocouple history was not recorded continuously during this interval; therefore, this test evaluates the temporal variation in the overall temperature output under the setpoint condition and does not independently quantify intrinsic sensor drift.

Given that the furnace employs a passive cooling mechanism by deactivating the heating elements—which inherently differs from its active heating process—the heating and cooling phases were analyzed independently. As shown in Figure 10, the FBG sensor exhibited good repeatability during the two heating and cooling cycles. The mean error, standard deviation, and variance between the two temperature measurements are presented in Table 6. During the 48-h monitoring shown in Figure 11, the peak-to-peak temperature-output variations of the three sensors were 3.89, 4.33, and 3.58 °C, respectively. The maximum sensor-specific peak-to-peak variation was therefore 4.33 °C, and the arithmetic mean of the three peak-to-peak values was 3.93 °C. Because no independent reference-thermocouple trace was recorded continuously over the same 48 h interval, these values include both furnace-temperature fluctuation and possible variation from the sensor and its packaging. Consequently, this dataset is reported as system output variation under an 800 °C furnace setpoint and is not used to independently demonstrate intrinsic long-term sensor stability or to rule out irreversible packaging-induced parasitic strain.

### 4.5. Bending Performance Testing of the FBG Sensor

**Figure 12:**
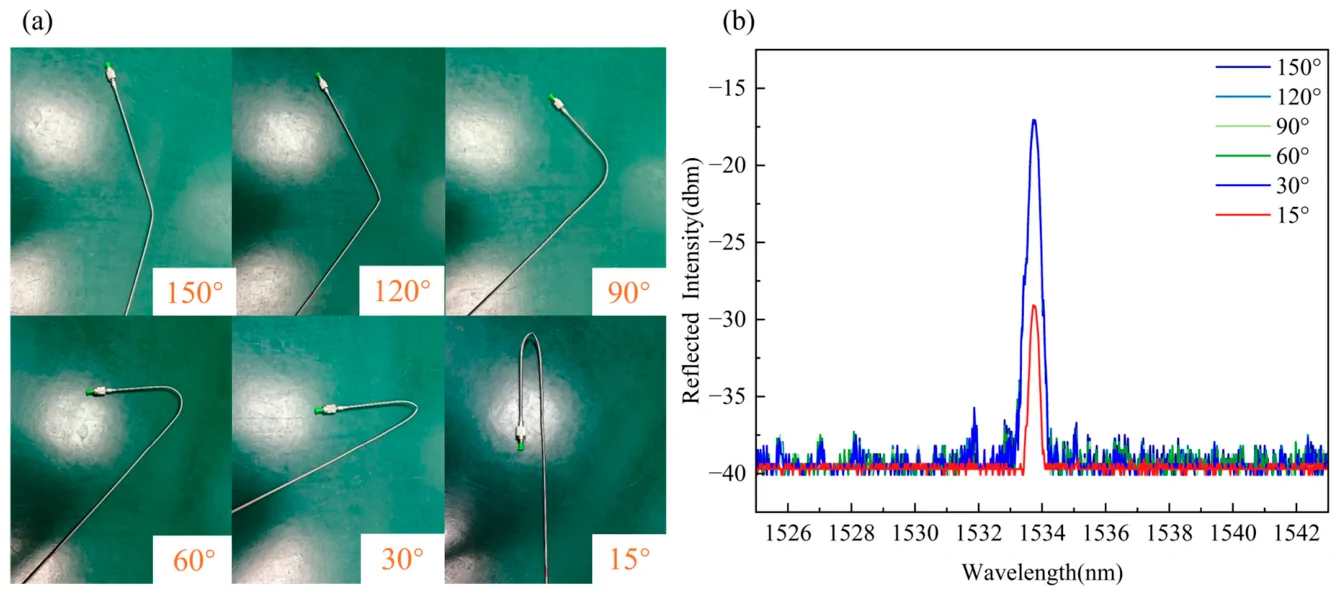
Bending test of the lead region after high-temperature experiment. (**a**) Fixed-angle bending test. (**b**) Optical spectra at various bending angles.

**Figure 13:**
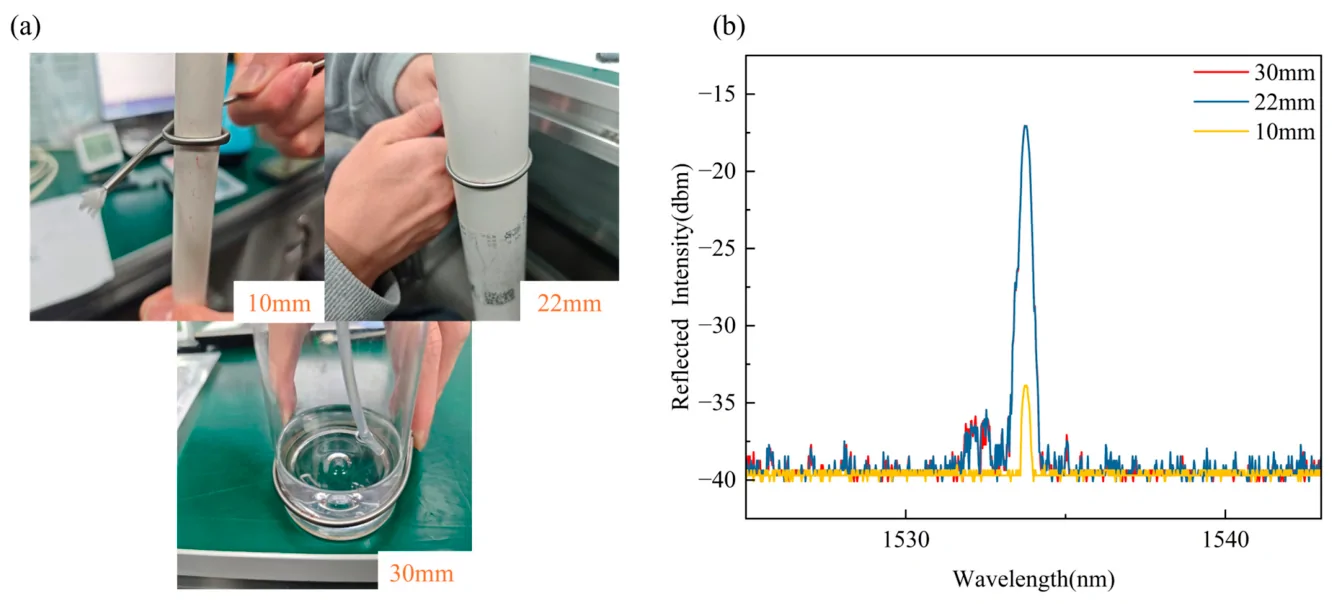
Bending test of the lead region after high-temperature experiment. (**a**) Schematic of the bending test. (**b**) Optical spectra at various bending radii.

**Figure 14:**
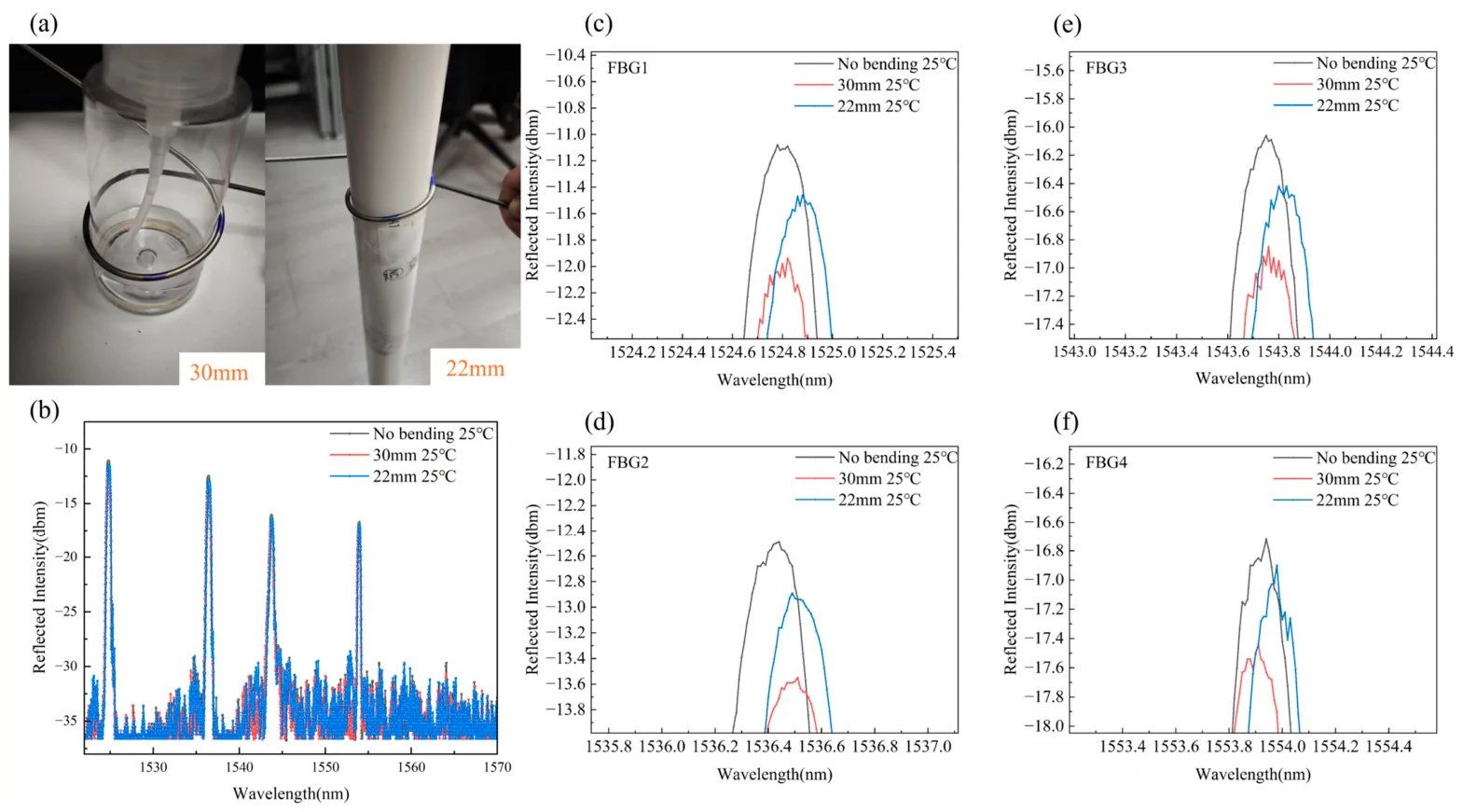
Bending test of the sensing region after high-temperature experiment. (**a**) Schematic of the bending test. (**b**) Optical spectra at various bending radii. (**c**) Enlarged views showing the effect of various bending radii of FBG1. (**d**) Enlarged views showing the effect of various bending radii of FBG2. (**e**) Enlarged views showing the effect of various bending radii of FBG3. (**f**) Enlarged views showing the effect of various bending radii of FBG4.

**Figure 15:**
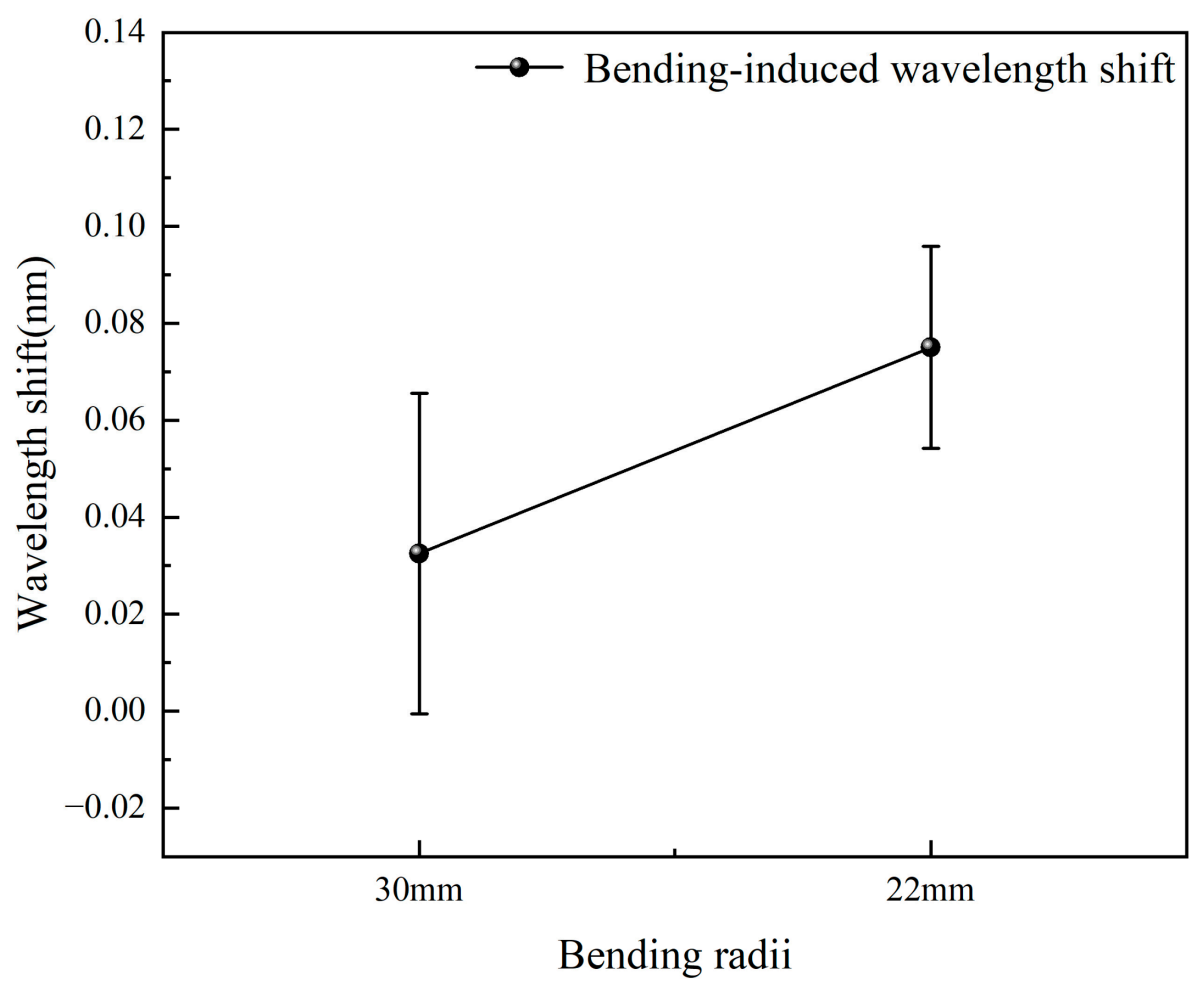
Error bars of the wavelength shift caused by different bending radii at room temperature.

**Figure 16:**
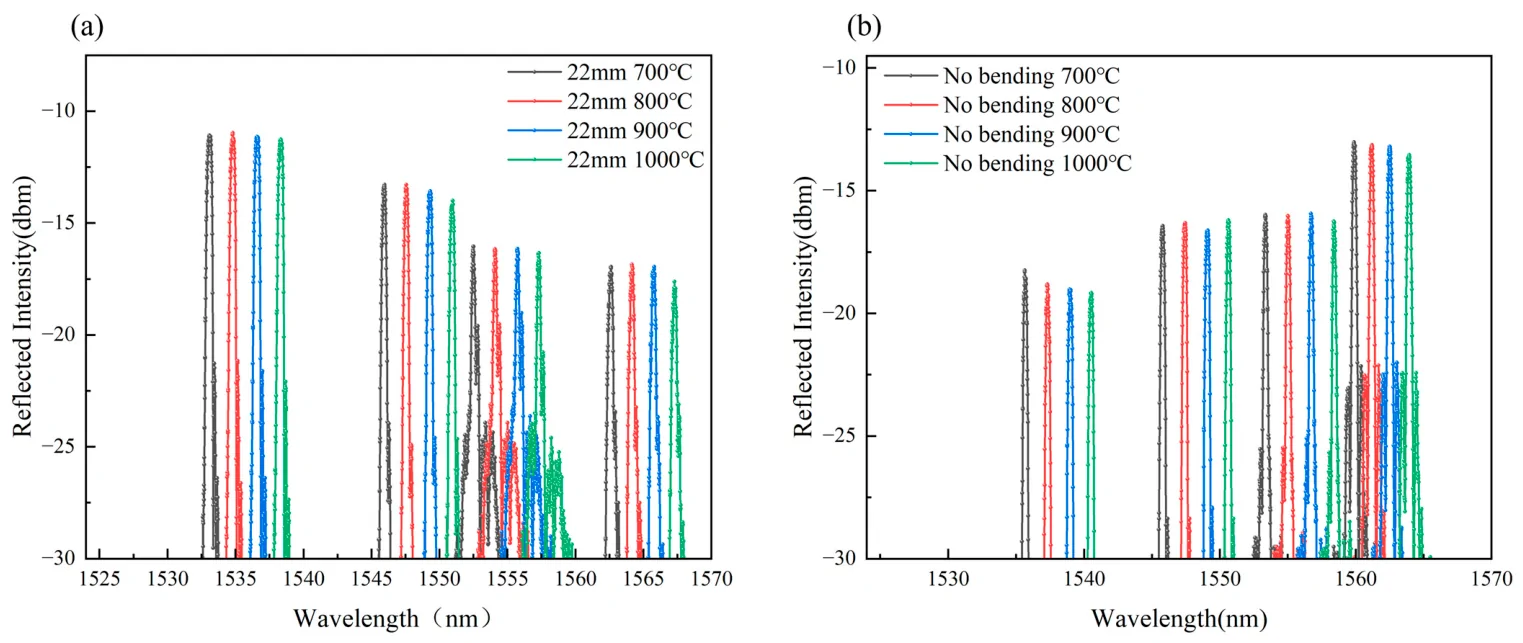
Comparison of the additional wavelength shifts induced by bending at various high temperatures. (**a**) Optical spectra of the sample with a 22 mm bending radius at different temperatures. (**b**) Optical spectra of the unbent sample at different temperatures.

**Figure 17:**
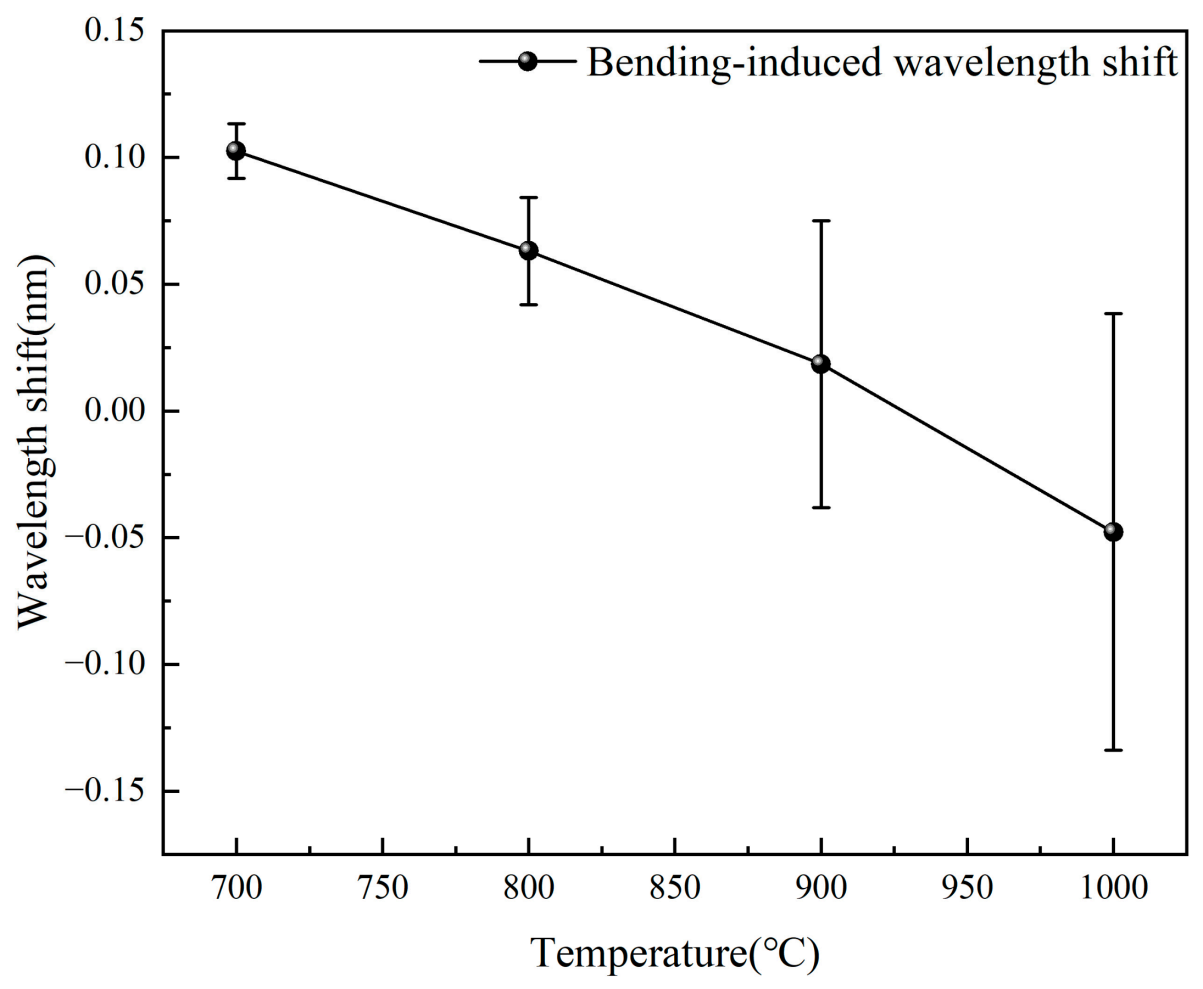
Unmatched-specimen wavelength-shift differences between the bent and unbent samples at different temperatures (22 mm bending radius). Error bars summarize the four FBG values and do not represent a propagated uncertainty of isolated bending strain.

In critical equipment within the aerospace and power generation sectors, such as gas turbine engines, internal spaces are highly confined, and installation pathways are often tortuous. Routing external sensors to critical temperature measurement points frequently requires navigating through multi-layered casing structures. Under these complex constraints, the flexibility and bendability of the sensor during deployment are of paramount importance. To investigate the ultimate bending performance of the sensor, we fabricated one single-grating sample and two samples containing four gratings. We refer to the array region containing four gratings (spaced 5 cm apart with a total length of 15 cm) as the sensing region, and the section behind the array responsible for transmitting optical signals as the lead region. The samples were first subjected to a high-temperature thermal treatment at 950 °C with an isothermal holding period of 8 h, followed by bending performance tests. As illustrated in Figure 12 and Figure 13, the subsequent lead region of the single-grating sample was subjected to bending tests at specific angles and fixed bending radii to determine the ultimate bending angle and bending radius of the lead region. Simultaneously, the sensing region of the four-grating sample underwent bending tests to evaluate its performance under specific bending radius conditions, with the relevant results shown in Figure 14 and Figure 15. Furthermore, a sample bent at room temperature and an unbent four-grating sample were placed together in a tube furnace for a heating experiment. This was conducted to evaluate the bending performance at high temperatures and to estimate an unmatched-specimen wavelength difference associated with the bent configuration. The experimental results are shown in Figure 16 and Figure 17.

As observed in Figure 12, when the bending angle of the lead region is between 150° and 30°, the sensor’s spectra perfectly overlap, maintaining a high SNR and thereby ensuring reliable peak wavelength demodulation. However, at the extreme bending angle of 15°, the spectral quality degrades significantly, the reflection peak intensity attenuates sharply, and the SNR becomes exceptionally poor, precluding accurate wavelength tracking. Similarly, in the fixed-radius bending experiments, when the bending radii are 30 mm and 22 mm, the spectra remain overlapped with robust SNRs. Conversely, reducing the radius to 10 mm causes a drastic drop in reflection peak intensity and SNR. Since angular bending is essentially a fold maintaining a certain bending radius, the underlying mechanisms for both phenomena are similar. Although the GH3030 alloy exhibits excellent bending performance, once the external bending exceeds a certain threshold, it undergoes structural deformation in the localized bending region. This irreversible deformation typically manifests as buckling or denting of the metal tube, and such localized collapse drastically compresses the internal volume of the sensor. This sudden compression of the internal space forces the quartz wool to exert radial stress onto the optical fiber, creating a tight localized bending radius at the hinge point. When this local bending radius falls below the critical radius for total internal reflection of the fiber, the original core-guided modes leak heavily into the cladding and convert into radiation modes, thereby causing a drastic drop in the reflection peak intensity. Consequently, for practical engineering layouts, a minimum bending radius of 22 mm is strongly advised.

As illustrated in Figure 14, bending tests were conducted on the sensing region containing the four FBGs (the grating locations are indicated by the blue markers in Figure 14a). As demonstrated by the optical spectra, both the 30 mm and 22 mm bending radii induce a redshift in the central wavelengths of the FBGs. Additionally, although the bending causes a slight decrease in the reflection peak intensity, the reflection spectra still maintain a good SNR. The specific wavelength shifts for each FBG under the 30 mm and 22 mm bending radii are detailed in Table 7.

At room temperature, the mean wavelength changes were 0.03 nm at a 30 mm bending radius and 0.08 nm at a 22 mm bending radius. Using the reported high-temperature sensitivity of approximately 16.3 pm/°C only as a scale conversion, these correspond to apparent-temperature equivalents of approximately 1.8 and 4.9 °C, respectively. The maximum 0.10 nm room-temperature shift at 22 mm corresponds to approximately 6.1 °C on the same scale. These values are not negligible relative to the unbent indication-error statistics and therefore must not be assessed only against the approximately 14 nm full-range thermal wavelength excursion. The room-temperature test demonstrates that all four peaks remain demodulatable at 22 mm; it does not demonstrate preservation of the unbent temperature accuracy at that radius.

Figure 16 compares a specimen held at a 22 mm bending radius with a different unbent specimen. Their wavelength-shift difference is not a rigorous isolation of bending strain because it also contains sensor-to-sensor differences in calibration, annealing history, packaging stress, spectral sensitivity, and local furnace temperature. The result is therefore reported as an unmatched-specimen differential rather than a pure bending-induced contribution. Both specimens retained demodulatable reflection peaks at high temperature, but quantitative accuracy in the bent state cannot be established from this comparison.

**Figure 18:**
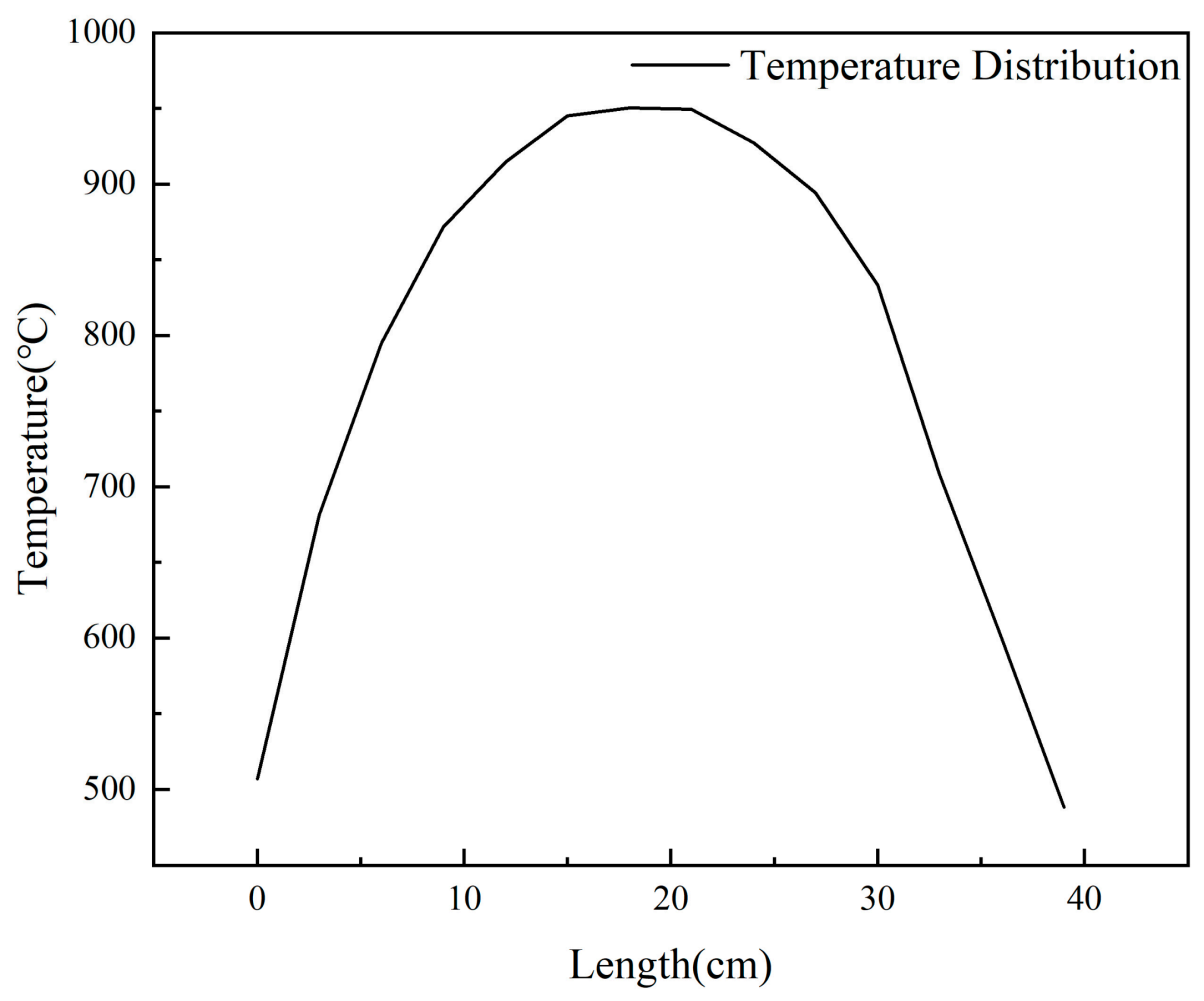
Temperature distribution of the tube furnace.

The unmatched-specimen differentials in Figure 17 include negative values at 900 and 1000 °C, consistent with confounding by furnace non-uniformity and specimen-specific response. The results of furnace non-uniformity are shown in Figure 18. The largest magnitude, 0.125 nm, corresponds to an apparent-temperature equivalent of approximately 7.7 °C when divided by 16.3 pm/°C. Because a matched before/after control at demonstrably equal temperature and the covariance information required for uncertainty propagation were not recorded, no uncertainty is assigned to a purported isolated bending contribution. The data support spectral operability of the bent package, but not strain-temperature decoupling or retention of the 2.02 °C unbent maximum indication error during bent operation. Matched calibration in the installed geometry or in situ compensation is required for quantitative bent-state measurements. Accordingly, bending of the sensing region should be minimized where uncompensated accuracy is important.

The room-temperature bending tests represent the mechanical routing step used during deployment, and the high-temperature spectra show that the installed bent samples remain optically demodulatable. These observations establish deployment feasibility only. They do not replace a matched bent-state calibration and should not be interpreted as proof that bending introduces no material temperature error.

## 5. Conclusions

In this paper, a flexible high-temperature fiber Bragg grating (FBG) sensor array with a quartz-wool/GH3030 dual-layer composite package is implemented and experimentally characterized as an application-specific extension of the compliant-inner/protective-outer packaging concept. Under the investigated conditions, this configuration supports measurements up to 1000 °C and spectral demodulatability at a demonstrated deployment bending radius of 22 mm. The package consists of an outer nickel-based superalloy tube and an inner quartz-wool layer. The inherent flexibility of the quartz wool is leveraged to cushion external mechanical stresses and constrain the posture of the optical fiber, whereby its overall bendability is significantly improved. Simultaneously, robust structural protection is provided by the outer superalloy tube, and the dynamic thermal response of the sensor is substantially accelerated by its elevated thermal conductivity at high temperatures. To optimize the fabrication process, a systematic multi-cycle annealing protocol at 950 °C for 24 h was established. Internal residual stresses are effectively relieved by this protocol, and the cyclic repeatability of the sensor at 1000 °C is drastically improved. It is demonstrated by experimental evaluations that a temperature-wavelength fitting correlation coefficient exceeding 0.9999 is achieved by the sensor array. Following thermal cycling tests, the average repeatability deviation is suppressed to ±0.35 °C. During 48 h output monitoring at an 800 °C furnace setpoint, the three sensor outputs exhibited peak-to-peak variations of 3.89, 4.33, and 3.58 °C, respectively. Because no independent reference-temperature history was recorded continuously during this interval, these values are interpreted as total system output variations rather than as isolated intrinsic sensor drift. Relative to the WRP-191 reference indications, the maximum absolute indication error was 2.02 °C and the mean absolute indication error was 1.25 °C; these comparison statistics are not presented as combined or expanded measurement uncertainties. Using a T99 endpoint, the FBG reached the endpoint 120 ± 40 s earlier than the S-type thermocouple in nine stepwise comparisons, although the 60 s thermocouple sampling interval limits this estimate. Three FBG transfer tests sampled at 1 s gave a T99 response time of 144.17 ± 2.75 s. Furthermore, a 22 mm deployment bending radius was demonstrated spectrally; quantitative accuracy at that radius required matched bent-state calibration or compensation. Overall, the experiments demonstrated the engineering feasibility of this specific packaging configuration for high-temperature monitoring in confined and curved installation paths; they did not establish universal performance superiority over sensor architectures evaluated under different temperature ranges, conditions, and metric definitions.

## Figures and Tables

**Table 1:** Standard deviations of the central-wavelength samples at each calibration point for FBG1-FBG4.

FBG	400 °C (nm)	500 °C (nm)	600 °C (nm)	700 °C (nm)	800 °C (nm)	900 °C (nm)	1000 °C (nm)
FBG1	1.52 × 10^−3^	7.42 × 10^−4^	1.23 × 10^−3^	9.99 × 10^−4^	1.03 × 10^−3^	3.73 × 10^−3^	8.62 × 10^−4^
FBG2	8.74 × 10^−4^	3.30 × 10^−4^	6.12 × 10^−4^	1.56 × 10^−3^	1.57 × 10^−3^	1.30 × 10^−3^	1.22 × 10^−3^
FBG3	6.99 × 10^−4^	3.67 × 10^−4^	2.46 × 10^−3^	2.77 × 10^−3^	3.78 × 10^−4^	6.19 × 10^−4^	3.16 × 10^−4^
FBG4	4.63 × 10^−4^	5.32 × 10^−4^	7.69 × 10^−4^	5.20 × 10^−4^	1.05 × 10^−3^	8.53 × 10^−4^	3.66 × 10^−4^

**Table 2:** Definitions and statistical status of the error-related quantities reported in this study.

Quantity	Reported Value	Definition and Interpretation
Maximum absolute indication error	2.02 °C	max|T_FBG_ − T_ref_| over the paired observations; not an uncertainty.
Mean absolute indication error	1.25 °C	mean (|T_FBG_ − T_ref_|) over the two heating and cooling cycles; not an uncertainty.
WRP-191 tolerance statement	0.2% FS + 0.5 °C	Manufacturer specification retained without conversion or numerical propagation because the configured FS and supporting calibration information were not documented.

**Table 3:** Descriptive comparison of source-reported high-temperature sensor metrics.

Year.	Author	Temperature Range	Reported Error Metric and Condition	Bending Radius	Response Time
2011	Barrera	25–1025 °C	NR; prior percentage-derived conversion removed	NR	50 s
2020 [19]	INFIBRA TEC	−50–500 °C	NR	150 mm	NR
2020 [20]	Kumar J	30–900 °C	±1.5 °C; reported for range up to 900 °C	NR	NR
2024	Khan R. Y. M.	27–700 °C	NR	NR	180 s
2024 [21]	Nan p	0–1400 °C	NR; temperature basis of prior value not verified	NR	250 s
2025 [22]	Wang Z	25–1700 °C	NR; temperature basis of prior value not verified	NR	NR
2026	This work	20–1000 °C; error evaluated at 500–1000 °C	MAE 1.25 °C; MaxAE 2.02 °C; two heating/cooling cycles	22 mm	144.17 ± 2.75 s; T99; 1 s sampling; n = 3

**Table 4:** Materials and diameters of the S-type thermocouple and FBG sensor.

	Outer Diameter	Sensor Material	Packaging Material
WRP-191	3 mm	Pt, Rh	Al_2_O_3_
FBG Sensor	3 mm	SiO_2_	GH3030

**Table 5:** Thermal conductivities of the nickel-based superalloy and alumina at different temperatures.

	20 °C	500 °C	800 °C
GH3030	15.1 W/(m·K)	20.9 W/(m·K)	25.1 W/(m·K)
Al_2_O_3_	30~35 W/(m·K)	11~12 W/(m·K)	6.9~7.1 W/(m·K)

**Table 6:** Error data from heating and cooling tests for FBG1–FBG4.

	Process	Mean Error (°C)	Standard Deviation (°C)	Variance (°C)
FBG1	Heating	0.60	0.76	0.57
	Cooling	–0.042	0.59	0.34
FBG2	Heating	0.56	0.84	0.71
	Cooling	–0.056	0.71	0.51
FBG3	Heating	0.45	0.72	0.53
	Cooling	–0.52	0.69	0.48
FBG4	Heating	–0.27	1.23	1.52
	Cooling	–0.30	1.04	1.09

**Table 7:** Wavelength shifts of the sensor under different bending radii relative to the unbent state at room temperature.

	30 mm	22 mm
FBG1	0.04 nm	0.10 nm
FBG2	0.07 nm	0.05 nm
FBG3	0.03 nm	0.08 nm
FBG4	–0.01 nm	0.07 nm

## Data Availability

Data underlying the results presented in this paper are not publicly available at this time but may be obtained from the authors upon reasonable request.
